# Surfer's ear

**DOI:** 10.1002/ccr3.929

**Published:** 2017-04-04

**Authors:** Yumi Okuyama, Akira Baba, Hiroya Ojiri, Tsuneya Nakajima

**Affiliations:** ^1^ Department of Internal MedicineTokyo Dental College Ichikawa General HospitalChibaJapan; ^2^ Department of RadiologyTokyo Dental College Ichikawa General HospitalChibaJapan; ^3^ Department of RadiologyThe Jikei University School of Medicine and University HospitalTokyoJapan; ^4^ Department of OtolaryngologyTokyo Dental College Ichikawa General HospitalChibaJapan

**Keywords:** Exostosis, external auditory canal, otolaryngology, surfer

## Abstract

Exostosis in external auditory canal is common among surfers. Common symptoms are decreased hearing or loss of hearing, ear infection, and/or plugging sensation. Repeated exposure to cold water is a key clinical history to suspect this condition. Based on symptoms and existence of infection, surgical removal of the exostosis is recommended.

An otherwise healthy 46‐year‐old man presented with hearing disturbance in his right ear of 1 day duration, after having gone surfing. Otoscope showed narrow external auditory meatus due to protruded mass (Fig. 1, arrows). A computed tomography (CT) showed multinodular bony mass protruding into external auditory canal (Fig. 2). There was no evidence of other bony injury or soft tissue involvement. This exostosis, also known as surfer's ear, most commonly occurs in individuals who have had repeated exposure to cold water 1, 2. Its prevalence among surfers is known to be 60–80% 1, 2. Surgical removal of the exostosis is performed when it becomes large enough to occlude the canal and/or infections develop 2.

**Figure 1:**
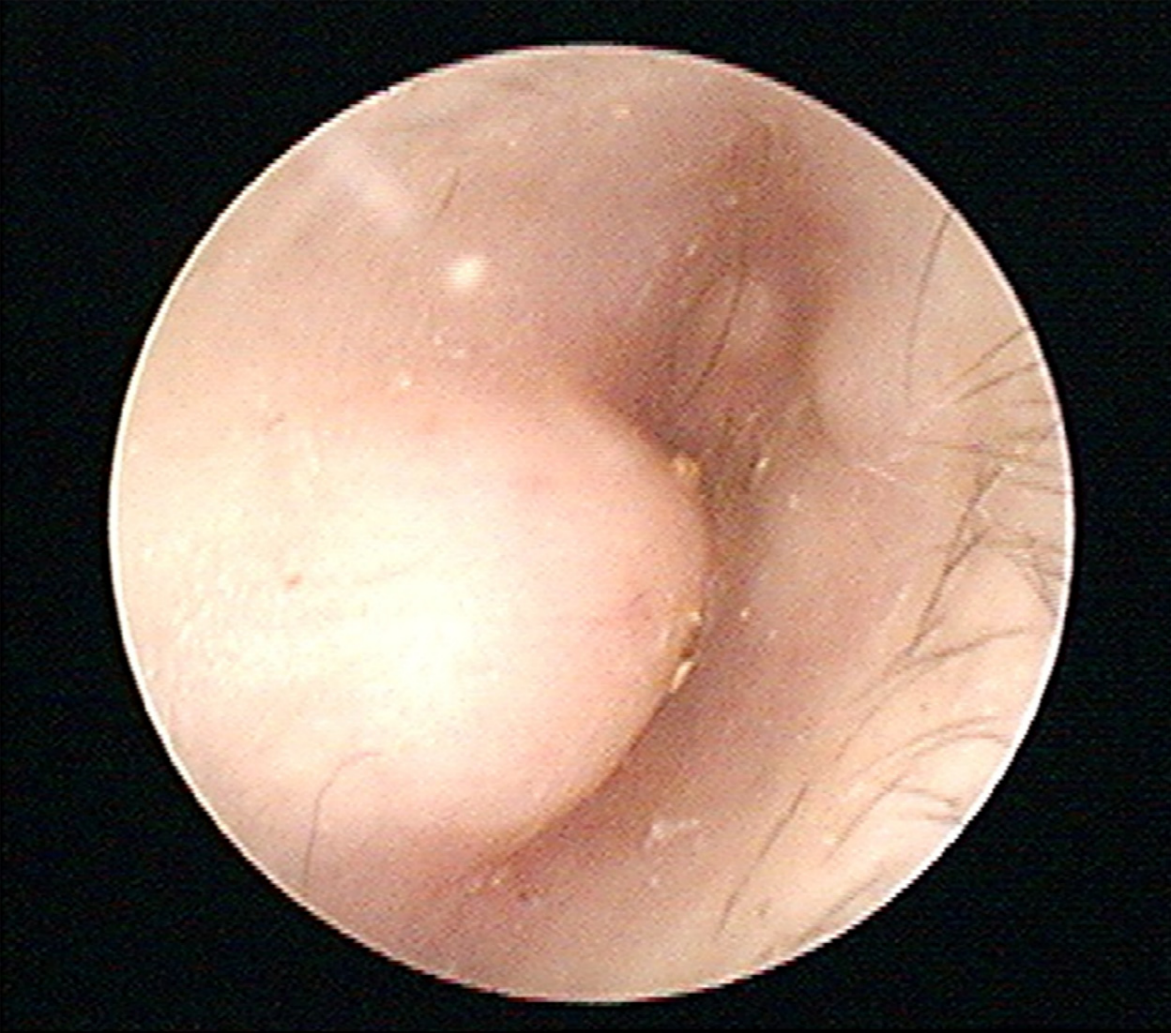
Otoscopic exam showing protruded mass.

**Figure 2:**
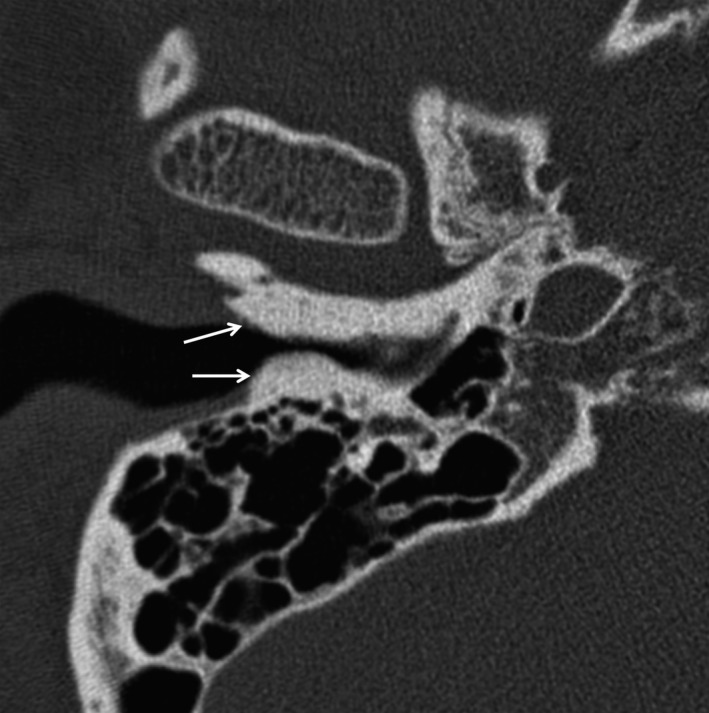
Computed tomography image of right temporal region.

Detailed history is important to help diagnose surfer's ear when patient is complaining of hearing disturbance.

## Authorship

YO: drafted the article. All authors participated in critical review and the revision of the articles. All authors gave the final approval of the article. All authors have accountability for all aspects of the work.

## Conflict of Interest

None declared.
